# Archetype-Based Modeling of Persona for Comprehensive Personality Computing from Personal Big Data

**DOI:** 10.3390/s18030684

**Published:** 2018-02-25

**Authors:** Ao Guo, Jianhua Ma

**Affiliations:** 1 Graduate School of Computer and Information Sciences, Hosei University, Tokyo 184-8584, Japan; 2 Faculty of Computer and Information Sciences, Hosei University, Tokyo 184-8584, Japan; jianhua@hosei.ac.jp

**Keywords:** archetype, scenario, persona, personality, personal big data, psychological model

## Abstract

A model describing the wide variety of human behaviours called personality, is becoming increasingly popular among researchers due to the widespread availability of personal big data generated from the use of prevalent digital devices, e.g., smartphones and wearables. Such an approach can be used to model an individual and even digitally clone a person, e.g., a Cyber-I (cyber individual). This work is aimed at establishing a unique and comprehensive description for an individual to mesh with various personalized services and applications. An extensive research literature on or related to psychological modelling exists, i.e., into automatic personality computing. However, the integrity and accuracy of the results from current automatic personality computing is insufficient for the elaborate modeling in Cyber-I due to an insufficient number of data sources. To reach a comprehensive psychological description of a person, it is critical to bring in heterogeneous data sources that could provide plenty of personal data, i.e., the physiological data, and the Internet data. In addition, instead of calculating personality traits from personal data directly, an approach to a personality model derived from the theories of Carl Gustav Jung is used to measure a human subject’s persona. Therefore, this research is focused on designing an archetype-based modeling of persona covering an individual’s facets in different situations to approach a comprehensive personality model. Using personal big data to measure a specific persona in a certain scenario, our research is designed to ensure the accuracy and integrity of the generated personality model.

## 1. Introduction

With the rapid advances in the digital explosion of data and connectivity, service and intelligence are growing in importance in the cyber-physical integrated hyperworld [1]. The amount of data created by this advance is sufficient to make a model of an integrated human individual feasible, enabling services more finely tailored to individual users. As one example, Cyber-I (cyber individual), a digital clone for a real individual (Real-I) featured with a unique and comprehensive personal description, has become an ideal framework for pervasive intelligence to better encompass the corresponding Real-I [2]. However, due to the dazzling yet unstructured abundance of information available, approaching Cyber-I by simply mining personal data is challenging, as a consequence of the lack of mature structure and high level of difficulty in computing a person’s mental state. Although a growth mechanism for Cyber-I modeling has been built in the laboratory [3,4], i.e., a Cyber-I model containing three growth mechanisms, namely Bigger, Higher and Closer, with a specially designed management system [5], Cyber-I modeling is still at the theoretical stage without any actual result. Because of the difficulty in ascertaining an individual’s mental state, the development of Cyber-I modeling is still at a vestigial stage. As using measuring aspects of personality to describe an individual’s mental make-up is mainstream in the field of Psychology, a mature theoretical system to do so exists within that discipline. Thus, the computing of personality based on insights achieved within that field has become a preferential aim for better Cyber-I modeling.

Personality is a psychological construct aimed at describing the wide range of humans’ habitual behaviors, cognitions and emotional patterns that evolve from biological and environment factors [6]. Currently, a lot of research is focused on or related to personality computing. In particular, the Big-Five (BF) personality trait theory, which contains a five-factor model (FFM), has been adopted as a theoretical foundation in leading research into personality computing, due to its long standing and widely validated research findings. According to the survey of Vinciarelli, personality computing can be classified into three areas based on three different approaches, namely Automatic Personality Recognition (APR), Automatic Personality Perception (APP) and Automatic Personality Synthesis (APS) [7]. APR aims at inferring traits through self-assessment, i.e., through a questionnaire. In contrast, APP focuses particularly on inferring the personality observers attribute to a given individual from proximal cues, i.e., through the judgment based on others’ perception. In addition, APS is in relation to the task of automatically generating distal cues aimed at eliciting the attribution of desired personality traits. This research has applied the Automatic Personality Recognition into personality computing due to its emphasis on inferring emotional and social phenomena from machine detectable behavioural evidence, which, in this case, refers to the emotional data gathered from surrounding devices. Thus, the personal data collection carried out in our previous work by a context-aware scheduling mechanism is highly suitable as a resource for Automatic Personality Recognition [8]. However, according to the survey mentioned above, current personality computing still relies on finding a correlation between a specific personal datum and the corresponding personality trait described in the five-factor model (FFM). These personality traits indicate broad features in terms of behavior, cognition and emotion. For example, people high in openness are motivated to seek new experiences and to engage in self-examination. Structurally, they have a fluid style of consciousness that allows them to make novel associations between remotely connected ideas. It is evident that this revelation of a person with high openness trait is too inaccurate to describe a specific human in different situations. For example, a person may show a poor ability of seeking new experiences when he feels nervous. Therefore, the comprehensive modeling of personality is urgently needed to achieve a more precise description of a human.

**Figure 1:**
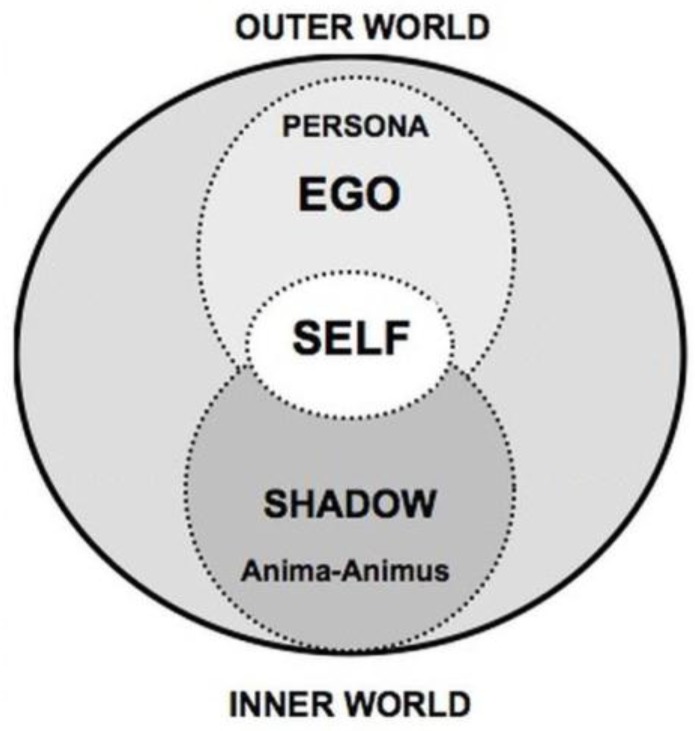
Jung’s theory of the “Self”.

According to the theories of Carl Gustav Jung (1875-1961), the concept of *persona,* as a kind of mask or social role, represents a compromise a person makes with the society concerning how he/she should appear to be [9] with an aim to conceal the true nature of the individual [10]. A person’s mental structure involves the persona and, as showed in Figure 1, the model that indicates the “self” can be described as consisting of three layers. As the outer layer of this structure, persona refers to the set of a human’s mental state in different situations or scenarios. The persona is also the connection between the external physical world and the inner mental world. In order to delve into a person’s inner world, the modeling of persona is therefore needed. Furthermore, the persona makes it possible to construct a digital personality model. In view of this, this research is aimed at achieving a comprehensive personality model from the modeling of persona.

As we know, diverse personae exist in any human’s social interaction. The modeling of sufficient personae for common situations is required to accurately describe a person. The heterogeneous data sources are selected accordingly, to provide the plenty of personal data (also named *Personal Big Data*). This personal big data covers all the personal data that could be gathered from daily life for human modeling, i.e., physiological data, the data from the Internet or the daily activity data. Such plentiful data provide the solid foundation for future persona modeling. In addition, the physiological data like emotion states that timely reflecting a person’s responses of activities would have high priority for the construction of persona, due to the warning from Jungians’ perspective that the researchers of persona should pay attention to hidden elements that may erupt if not made conscious [11].

Although the personal data is more than sufficient, it seems hard to construct a person’s persona without any persona forms. Fortunately, a prototype persona could be derived from the twelve typical human types, or *archetypes*, as outlined in Jungian psychological theory. The concept of an archetype was conceived by Carl Jung [12] in his work on the collective unconscious as a typical character to whom an observer might emotionally resonate. Collective unconscious embraced impersonal, universally shared, fundamental characteristics of humanity that he referred to as primordial images or archetypes [13]. Based on myths, legends and esoteric teachings, archetypes form part of the individual’s unconscious mind [14]. That is to say, a specific persona in a certain scenario would take on the aspect of one or more archetypes. Such archetypes could be detected from different scenarios. For example, when playing card games, a person may exist as the archetype Hero, referring to his desire to win the game. However, the person could also show the archetype Lover, as he/she may want to help a partner in certain games. Hence, the first target of this research is to create a persona derived in one specific diverse scenario existing as a combination of different archetypes.

In order to acquire a comprehensive description of a human, the second objective of this research is to model a series of personae representing a comprehensive description of a certain person, or personality. One of the groupings of personae is called *facet persona* (FP), in which the term ‘facet’ in the psychological field refers to a specific and unique aspect of a person’s mental state or boarder personality trait, such as the mental state demonstrated on the Internet [15]. Specifically, each person could be modeled to one special description called a *primary persona* (PP), denoting the primary personality state in which a person presents themselves.

The remainder of this paper is organized as follows: the following section introduces related studies and their relevance to this research. Section 3 provides an overview of each function for personality modeling. The personal data collection from heterogeneous data sources is explained in Section 4. Scenario detection from event location, contact person to user behavior is given in Section 5. Section 6 describes the method for the characterization of the twelve archetypes. Subsequently, the archetype-based modeling of persona is given in Section 7. As the proposed personality model, the computing of facet persona and personality are explained in Section 8. Experiments and discussion are presented in Section 9. Conclusions and future work are outlined in the last section.

## 2. Related Works

Over a brief period of time, computing has come to focus on personal life through the increasing prevalence of smart devices. Subsequently, a great deal of research is being conducted into collecting personal data. Chittaranjan et al., investigated behavioral characteristics derived from rich automatically extracted smartphone data [16]. Daniel et al., developed a wearable sensing platform to automatically capture individual and collective patterns of behavior, which is able to be used to predict further user behavior [17]. In addition, context-awareness techniques have become applicable to personal data collection. One salient example of this is our previous research into context data detection and data collection scheduling in a smartphone-based client-server system. A systematic wearable data collection schedule was implemented with a corresponding context-aware engine to handle different contextual information. Accordingly, in this research a scenario and a persona are detected separately, based on previously collected wearable data, including GPS data, acceleration data, and sleep data, in order to collect information in terms of user behavior, location, and even communication.

With the increasing usage of the Internet, there is a pressing urgency to depicting user behavior in social networking, which has led to the creation of user models derived from those users. *User Persona* as one user modeling technique to be used in product design and Human Computer Interaction (HCI), was first put forward by Cooper in 1999 [18]. Different from the description of persona in psychology, “personae used for the product design refer to fictional detailed archetypical characters that represent distinct groups of behaviors, goals and motivations observed and identified during the research phase”, as defined by Goodwin [19]. To create a fictional typical user in possession of rich detail for specific requirements, Wu, et al., proposed a method that could swiftly transform a customer’s needs into a persona based on analysis of typical product utility [20]. To accurately create a persona, Fakinlede, et al. designed a well-defined robust psychometric model for defining and effecting a persona and depicting its functional role [21]. AlMaliki, Ncube and Ali tried to build personae by adopting feedback from generated personae instead of from real users to improve software. Analysis or estimation may become another issue for research into persona [22]. What’s more, taking ontology as a conceptual vocabulary, Salma et al., modelled and stored personae with a Resource Description Framework (RDF), a descriptive language [23]. Similar to the research above into persona modeling, in this research the prevalent persona model is also applied, especially in its elements. In addition to the research focusing on persona modeling, Phuong, et al., estimated the motivation and learning strategy of students for the improvement of an educational course [24]. Additionally, Friess explored incorporating personae into the heuristic evaluation of websites [25]. Tu et al. have used cluster analysis to examine the persona development [26]. Specifically, the Persona Creation and Usage Toolkit is adopted to cluster nine categories in terms of the user’s biographic background, user goals, knowledge, emotional characteristics of usage and etc. Similarly, this research selects two of the Big Five personality traits, the *Emotion Stability* and the *Extroversion/Introversion* to represent the calculated personality. Taking advantage of persona in online health communities, Huh et al, annotate four personae from the clustering of the usage behavior pattern of online communities based on a large amount of survey [27]. Nevertheless, these four personae in Jina’s research, namely the Caretakers, Opportunists, Scientists and Adventurers, are only suitable for clustering the users belonging to a certain group with a specific purpose. In contrast, this research aims at a series of stable personae from different scenarios. To ensure the general applicability for universal situations, each persona is comprised of one or more of Jungian’s archetypes. In conclusion, the related modeling of persona by Cooper just regards the persona as a fictional user upon its application. The resulting persona is analogous to the relationship between a brief resume and an actual person. In contrast to the persona research described above, one feature of persona modeling in this research is the creation of a persona based on twelve human archetypes. The digitalization of a persona based on Jungian psychological theory makes it possible to construct a persona that describes a person accurately and comprehensively. Another feature differentiating this research from previous research is the application of the modeling through facet personae. This research mainly aims to mine personal traits for comprehensive personae sufficient for Automatic Personality Recognition.

Jungian’s twelve archetypes have been used by many other studies as they involve the universal goals, motivations and desires in an individual’s daily life. Bechter et al. have identified the correlation between Jungian’s archetypes and personality traits based on the statistics of 102 executive MBA students [28], suggesting the usability of the archetypes. Similarly, on the basis of these fundamental archetypes, our research builds the persona modeling after conducting a timely questionnaire with the testers’. In addition, Munteanu et al. have found the correlation between archetypes and Myers-Briggs Personality features [29]. Besides, Pera, Viglia and Furlan have calculated the correlation among several variable characters including the archetypes, phases and covariates [30]. Research in relation to Jungian’s archetypes has indicated the application possibility of archetypes for human modeling.

**Figure 2:**
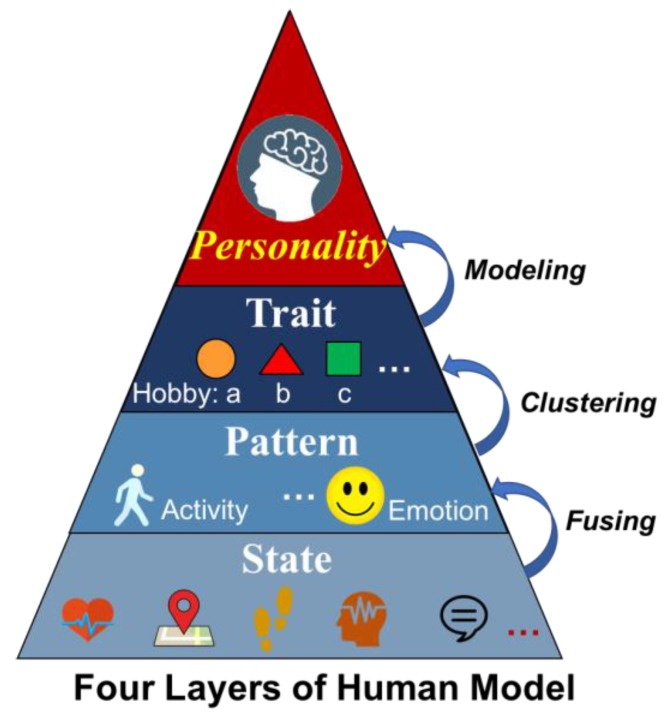
Human model description.

One issue for Cyber-I modeling is the accurate and comprehensive computation of an individual’s personality. Generally, the human model can be classified into four layers, as shown in Figure 2. The bottom layer of human model involves various human states, such as the physiological state, i.e., heartrate and brain activity, or the behavior state, i.e., running, walking, or talking, etc. For the second layer, the person’s pattern such as their activity pattern or emotion pattern are described. More abstractly, the third layer shows the person’s traits in different aspects, for example taking part in a hobby such as watching movies, or their working style. These three layers covers the aspects of a human from the concrete to the abstract. However, the top layer is the modeling of a human’s psychological state, namely personality. To the present day, there has been a great deal of research into personality computing based on the widely used “Big Five” personality trait theory depicting users’ features from five different aspects. Guntuku et al, create a personality model from the perspective of user favorites [31]. Vinciarelli surveyed personality computing in terms of measuring, perception and evaluation [32,33]. According to this survey, Mohammadi and Vinciarelli attempted Automatic Personality Recognition based on users’ prosodic features [34]. In this research, the research methods of personality recognition have also been adopted as one aspect of personality computing. The difference lies in perception being based on personae for the general depiction of an individual’s pattern in all aspects of daily life, instead of merely being derived from a single or limited number of certain situations.

## 3. System Overview

**Figure 3:**
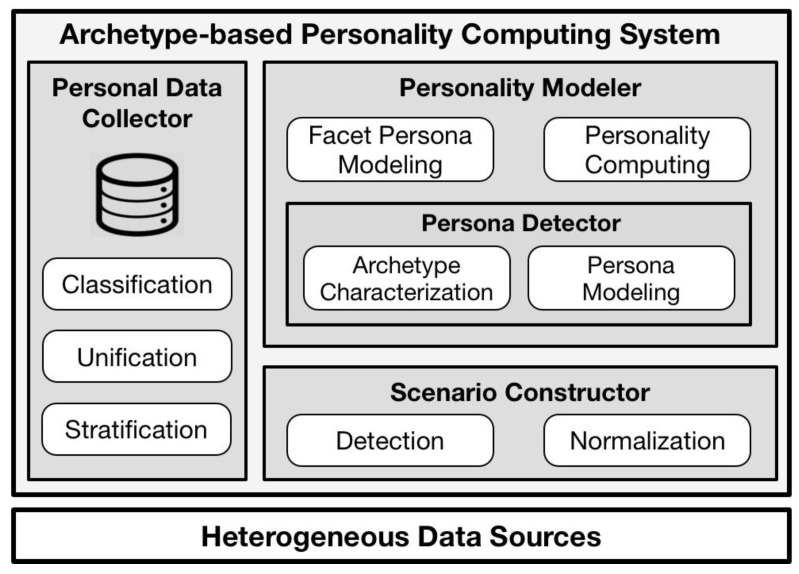
Archetype-based system for personality computing.

In our previous work, a conceptual model was provided to show the main steps of personality computing, and to provide evidence for the utility of persona. However, such a model was unable to provide enough stability in long-term running from data collection to personae generation. To achieve a comprehensive and stable approach to personality computing, an *archetype-based personality computing system* (ABPC system) has been built accordingly, as shown in Figure 3. The ABPC system consists of a personal data collector, a scenario detector, and a personality modeler. The details of the heterogeneous data sources and the personal data collector are introduced in sub-section one, and the function of the scenario detector is shown in sub-section two. As the core part of the personae modeler, the persona generator is demonstrated, particularly in sub-section three. Finally, sub-section four illustrates the function of the personae modeler.

### 3.1. Personal Data Collector

Personal Data is the foundation of human modeling. As mentioned in Section 2, personal data refers to the kind of data which relates to or is generated by a real person. For example, GPS data from a smartphone shows a person’s location, heartbeat rate data from an iWatch reveals a person’s health condition, and the browser data from Chrome can not only tell us a person’s favorite website types, but also shows the exact time of his/her activity surfing the internet. To describe a person’s condition and activity as accurately as possible, it is necessary to choose data sources that can provide diverse personal data. Therefore, *heterogeneous data sources* are selected correspondingly, which comprise of a social network, wearable devices, a smartphone and ambient devices to provide the raw data that can cover the major aspects of a person’s daily life.

As shown on the left in Figure 3, the personal data collector contains three functions; *data stratification*, *data unification* and *data classification*. As raw data collected from data sources is heterogeneous and can be used directly for further modeling, this data should first be classified. For example, the GPS data from the smartphone and the breath data from smart watch are collected at the same time. Clearly, the GPS data can only indicate a person’s location, and can’t be used for emotion detection. Therefore, the data stratification function is in charge of such data usage classification.

Due to the heterogeneity of raw data, data types vary from source to source. One key problem is that there is no unique method to handle the processing of all the various types of data. Actually, some kinds of data may involve deeper-level information that needs to be further excavated. For example, variation in heartbeat rate could also indicate a person’s mental state, i.e., if they are relaxed or nervous. Thus, the data unification function is intended to mine the extra information from the raw data for further processing, i.e., scenario construction and persona modeling. As the last function, the classification function inside the personal data collector is intended to bind the different types of data with the same time section. The detailed explanation of the functions from personal data collector is drawn in the next section.

### 3.2. Scenario Constructor

In Human-Computer Interaction (HCI), scenario refers to the context of an activity so that it can be discussed for system refinement. For example, chatting with friends on WeChat or sharing something on Moments could be regarded as two different scenarios in terms of WeChat APP. Obviously, the scenario used for HCI, also named an application-oriented scenario, could separate the user activities on a certain application into several situations. However, the scenario in terms of personality computing mainly has two features. Firstly, the heterogeneous data sources in this research guarantee that data is gathered in diverse ways, i.e., from wearables, smartphones or the internet, so that the scenario is able to involve the human state in all aspects of life but not exclusively as activities on a single HCI system. Therefore, the function *scenario construction* inside the scenario detector is to gather all the possible data according to the person’s activity. The key point is how to ensure the data highly related to a specific scenario. Secondly, the main goal of the scenario construction in this research is to categorize the environment for the mental state modeling of a specific subject. Such a scenario is named the personality-oriented scenario. Thus, the *scenario normalization* function inside the scenario detector is to normalize the personal data into several aspects, i.e., time, location and activity, for further personality computing. The process of scenario detection and key methods for scenario normalization are discussed in Section 5.

### 3.3. Persona Detector

As defined by Swiss psychiatrist Carl Jung, a persona was the stable social face the individual presented to the world. Persona is similar to a role, which describes each human’s psychological status in in terms of the person’s interaction with society. For example, a person may present different personae when interacting with different people, i.e., chatting with parents, going out with friends, or working with their boss. We normalized the interaction with others into several kinds of scenarios. Therefore, such mental variation is the unconscious performance by our inner mind in different scenarios. To research deep into a person’s inner mind, one straightforward way is to research his/her persona. However, the persona currently is simply a psychological description and hasn’t been digitally modelized yet. The persona as understood in psychology is difficult to use in personality computing. Therefore, the main target of *persona detector* is to digitalize the psychological persona into a computable digital persona form.

As we know, humans are complex and may present a different persona in each different scenario. Hence, to digitalize the persona, the core issue is to seize on the invariable features present in a human’s social activity. According to the Jung’s theory of personality, archetypes, which consist of twelve different aspects act as the prototype of a human’s psychological pattern and exist within the human mind. Actually, a human may present different archetypes in different scenarios. Hence, the *archetype characterization* function inside the persona generator is needed for the further *persona modeling*. A detailed explanation of archetype characterization and persona construction is explained in Section 6 and Section 7, respectively.

### 3.4. Personality Modeler

Although a person may present different personae in the different scenarios, for a mature individual, his/her persona in various scenarios is relatively stable. The *Personality modeler* is to depict a person according to all his personae present in different scenarios. Firstly, to describe a particular person, the characterization of personae with different aspects is necessary. For example, when together with friends, a person could be summarized as an honest person who is willing to help others but also wants to be a hero. Hence, the *facet personae modeling* function inside the personality modeler is to characterize a person’s persona due to some universal facet, i.e., people, activity, etc. Secondly, the *personality computing* is to give a summary of a person according to all his facet personae. The detail of modeling of personality modeler is explained in Section 8.

## 4. Personal Data Collection

In this section, heterogeneous data sources with two features are discussed in the first sub-section. Due to such duality of features, the main step of data collection is proposed accordingly, and is explained in the second sub-section. As the steps of data collection, personal data stratification, personal data unification, and personal data classification are clarified in the last three sub-sections.

### 4.1. Heterogeneous Data Sources

With the popularity of the Internet and Internet of Things (IoT), more and more personal data is accessible. In this research, the smartphone, wearable devices, ambient devices, and the Internet are selected to provide plenty of data for personality computing, and Table S1 shows all the personal data types and their sources. Due to their heterogeneity, three features of these data sources hindered scenario construction and persona modeling. These three features of heterogeneous data sources are shown below.

- *Dynamic Data Availability*: For a particular type of personal data, the availability is limited by the user’s behavior, user’s location, and the device battery. Therefore, the scenario construction and persona modeling function are facing the limitations of dynamic data sources.
- *Multiple Data Sources*: For a particular type of personal data, the data is provided by multiple sources, such as the smartphone and smartwatch both providing GPS data. Therefore, the selection of the appropriate data source is needed.
- *Multilevel Data Sources*: The data level varies from type to type. For example, the smartwatch could provide heart rate data every minute, and such data could provide an accurate personal emotion recognition. However, the sleep data provided by a smartphone which just indicates the users’ sleep condition hourly can only be used for approximate personal activity recognition. Hence, only part of the data is prepared for scenario construction, and other parts for persona modeling.

### 4.2. Flow of Personal Data Collection

**Figure 4:**
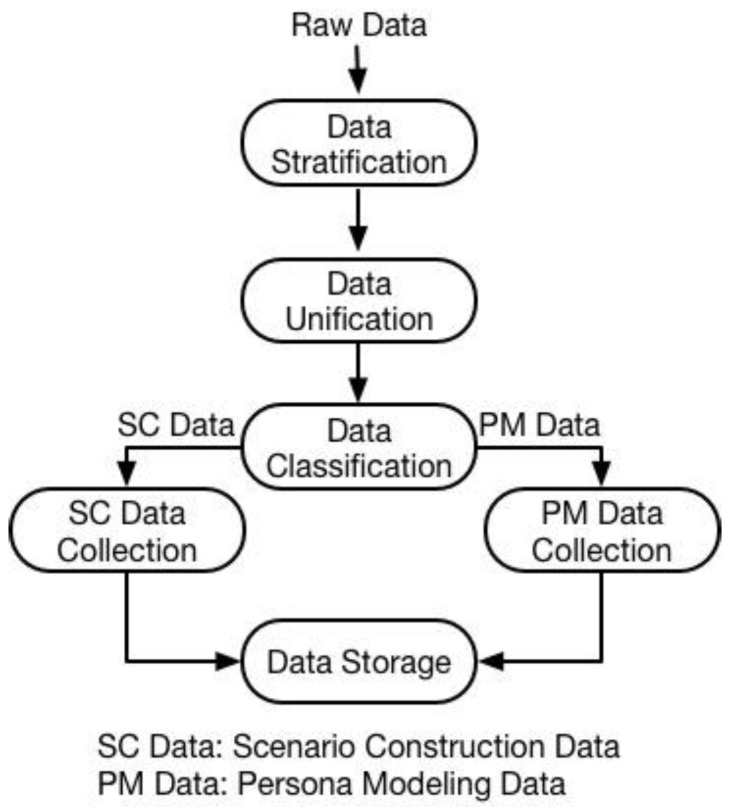
Data collection flow.

Due to data sources dynamically changing, collected raw data should be processed frequently when used for further scenario construction and persona modeling. The data collection flow is shown in Figure 4.

The data collection mainly consists of three sub-functions. They are data stratification, data unification, and data classification. Due to the dynamic change involved in the data sources and also the diversity of data types, the ABPC system is unable to categorize the exact type and quantity of collected personal data in advance. Taking advantage of such raw data for further modeling without any processing may lead to inadequate or even incorrect data utilization. For example, the step data provided by smart shoes could easily describe a person’s walking speed and the step frequency of their gait thanks to the high quality of the data and a sufficient data sampling frequency. However, the data quality will deteriorate, and such step data only indicate that a user is moving or not during periods of time when the smart shoes are not available, and the only data source is a smartphone.

To reduce incorrect data utilization, an initial data classification is necessary. Hence, the data stratification is to separate the data into three layers according to the temporal raw data type, so that different data utilization functions, i.e., scenario construction and persona modeling, can use different layer of data. To reduce inadequate data utilization, data unification is developed accordingly. The main purpose of data unification is to first mine one layer of data, and then generate new data in different layers. Such a process is named the second data collection. Finally, the data classification will classify the data into two groups for scenario construction (SC) and persona modeling (PM) separately. The detail of three sub-functions is explained in the following three sub-sections, respectively.

### 4.3. Personal Data Stratification

**Figure 5:**
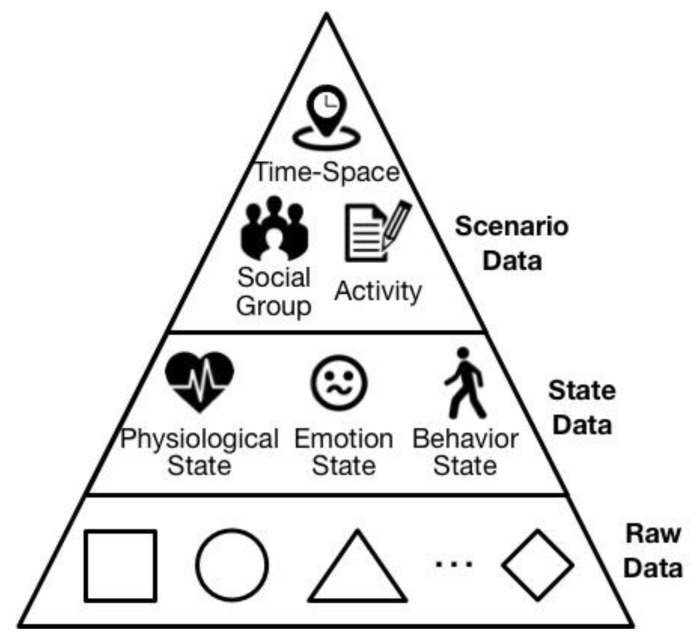
Demonstration of data stratification.

Being the first process of data collection, the data stratification function classifies the data into three layers, and its demonstration is shown in Figure 5.

The main use of collected personal data is for further scenario construction and persona modeling. Due to the differences in the elemental composition of scenario and persona, their requirements for personal data are correspondingly different. Concretely, scenario construction needs data that indicates the basic elements of a scenario, i.e., time, location, type of activity and who is involved. Hence, such data, named scenario data, consists of the top layer of personal data. In contrast to the scenario data, the state data is for the comprehensive description of a person’s state within a concrete scenario, as shown in the center of Figure 5. Because scenario data is limited to showing the rough activity type, i.e., walking, playing, or chatting with others, such data is not rich enough to provide description of a personal state. Hence, state data contains a person’s records of all of the states and its variations in terms of physiology, emotion, and behavior. Consequently, during the same scenario, the differences in state data from person to person reflect the uniqueness of the individual. Aside from the two kinds of data above, other kinds of data are classified into the raw data, as is shown in the bottom layer of Figure 5. Raw data is data that cannot obviously indicate the scenario elements or human state. However, by further digging, such data may indicate some hidden information which contributes to the scenario data and state data. For example, the voice records on a person’s smartphone may be detected by machine-learning, and may identify a person or people in the immediate vicinity of the smartphone carrier. The process of obtaining the scenario data and state data by mining this raw data is called personal data unification. The details of personal data unification are explained in the next sub-section.

### 4.4. Personal Data Unification

During the data collection, the majority of collected data is regarded as raw data, and only a few data that contain the apparent scenario data or human state data can be classified into the top layer and second layer in Figure 5. Hence, as mentioned in the last sub-section, personal data unification mines the hidden information from the raw data as thoroughly as possible and unifies the data with the requirements of scenario construction and persona modeling. Two different methods are provided for personal data unification, namely scenario data unification and state data unification. The core method of these two unifications relies on calculating the correlation between the specific personal part of raw data and the target data. Then such chunks of raw data can be used for target data verification, or even for data mining. One thing that should be clarified is that the correlation between raw data and target data varies from person to person. Therefore, the correlation will be calculated specifically to ensure accurate results and full usage of the raw data in each subject’s case.

### 4.5. Personal Data Classification

Although the data is separated into three layers by the data stratification function, to analyze a person’s state in a certain scenario, an association between scenario data and state data is necessary. Concretely, the state data should be weighted according to the scenario a person is involved in. Therefore, the personal data classification as the final part of data collection binds the scenario data and state data according to the scenario elements. For example, the state data would be classified according to different time and location. One thing that should be emphasized is that the time factor is indispensable. For example, the personal data would be classified according to linked factors such as time and location, or time and activity.

## 5. Scenario Normalization

**Figure 6:**
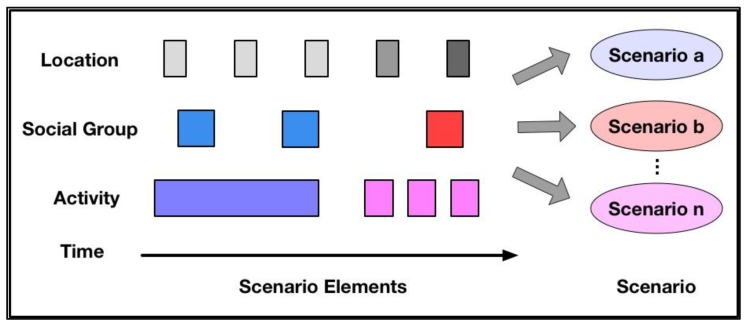
Overview of scenario normalization.

As described in section three, a scenario serves as a bridge for modeling personality from heterogeneous personal data. An overview of scenario detection is set out in the next sub-section. Exactly as shown in Figure 6, scenario elements description, the process of scenario normalization and its key methods are illustrated separately in the last three sub-sections.

### 5.1. Description of Scenario Normalization

Figure 6 provides an overview of scenario normalization. The labels of scenario elements are shown on the left in Figure 6. They are location, social group, and activity. In the middle of Figure 6, each small square refers to a scenario element corresponding to its label on the left. From left to right, array of squares is arranged in time order. For a certain square array, for example, a square with a different color refers to a different activity, and the width of each square represents the duration of such activity according to the personal data. The scenario normalization function detects and generates a set of normalized scenarios from the scenario data over a period of time. The formulation of scenario elements is illustrated in the next sub-section.

### 5.2. Scenario Element Description

In this sub-section, the location, social group and activity are clarified separately with the following formulae.

#### 5.2.1. Location

As an essential factor of a scenario, event location can be recorded according to the GPS location coordinates. The classification is shown in Equation (1):

(1) $$\begin{array}{l} L=\left\{l_{1},l_{2},\ldots ,l_{n}\right\}\\ l_{k}=\;\left({\mathrm{Lng}}_{k},{\mathrm{Lat}}_{k}\right), \end{array}$$

where each location element of $l_{k}$ belongs to the location set L, and $l_{k}$ is comprised of longitude ${\mathrm{Lng}}_{k}$ and latitude ${\mathrm{Lat}}_{k}$. The location set L can be clustered with the DBSCAN [35] algorithm (Density-based Spatial Clustering of Applications with Noise). The clustering process is shown in Equation (2):

(2) $$L_{\mathrm{Cluster}}=\mathrm{DBSCAN}\left(L,\varepsilon ,\mathrm{Minpts}\right),$$

where a fixed parameter ε and the minimum number of points, Minpts, have to be specified separately. The DBSCAN algorithm is well suited for location detection, because it is unnecessary to specify the size of the location set in advance.

#### 5.2.2. Person in Social Group

The person in social group means all the persons involved in a certain scenario. For example, a person’s friend could be regarded as one person in a social group when they are conversing. The classification is shown in Equation (3):

(3) $$\mathrm{SG}=\left\{p_{1},p_{2},\ldots ,p_{n}\right\},$$

where each person $p_{k}$ belongs to the social group SG.

#### 5.2.3. Activity

The third scenario element is the activity. In previous research, user’s activity was detected from wearable devices, a computer logger and the Internet data, such as that derived from checking email, surfing the web, chatting via WeChat, sleeping, walking, etc., and is shown in Equation (4):

(4) $$\mathrm{ACset}=\left\{{\mathrm{ac}}_{1},{\mathrm{ac}}_{2},\ldots ,{\mathrm{ac}}_{n}\right\},$$

where each user’s activity is covered in the activity set ACset.

### 5.3. Process of Scenario NorMalization

As mentioned above, the scenario generation functions are based on three essential factors. Therefore, it is critical to classify a new scenario and similar scenarios according to these three factors as accurately as possible. In order to present the algorithm for scenario generation, scenario is classified as shown in Equation (5):

(5) $${\mathrm{SC}}_{\mathrm{set}}=\left\{{\mathrm{sc}}_{1},{\mathrm{sc}}_{2},\ldots ,{\mathrm{sc}}_{n}\right\}$$

where each scenario ${\mathrm{sc}}_{k}$ belongs to the scenario set ${\mathrm{SC}}_{\mathrm{set}}$.

The algorithm for scenario generation is presented in Algorithm 1, in which the user’s activity ${\mathrm{ac}}_{j}$, location $l_{j}$ and social group $p_{j}$ are measured separately. If ${\mathrm{ac}}_{j}$ and $l_{j}$, or ${\mathrm{ac}}_{j}$ and SG exist simultaneously, such a scenario can be classified with a similar scenario ${\mathrm{Scenario}}_{\mathrm{similar}}$. Otherwise, such a scenario has to be classified as a new scenario ${\mathrm{Scenario}}_{\mathrm{new}}$.

**Algorithm 1:** Algorithm for Scenario GenerationInput: location $l_{j}$, person in social group $p_{j}$, user activity ${\mathrm{ac}}_{j}$, ${\mathrm{Scenario}}_{\mathrm{new}}$, ${\mathrm{Scenario}}_{\mathrm{similar}}$Output: Scenario ${\mathrm{sc}}_{j}$1: if ${\mathrm{ac}}_{j}\notin \mathrm{ACset}$ then2:  ${\mathrm{sc}}_{j}$ = ${\mathrm{Scenario}}_{\mathrm{new}}$3: else if $l_{j}\notin {L\;\mathrm{or}\;p}_{j}\notin \mathrm{SG}$ then4:  ${\mathrm{sc}}_{j}$ = ${\mathrm{Scenario}}_{\mathrm{new}}$5: else6:  ${\mathrm{sc}}_{j}$ = ${\mathrm{Scenario}}_{\mathrm{similar}}$7: end if

### 5.4. Key Methods for Scenario Normalization

There are two main issues for scenario normalization. The first one is how to ensure the scenario start time/end time and its elements are relatively accurate. The second one is how to classify multiple scenarios at the same time. There are three main methods, and they are explained in this sub-section.

#### 5.4.1. All-Elements-First Algorithm

**Figure 7:**
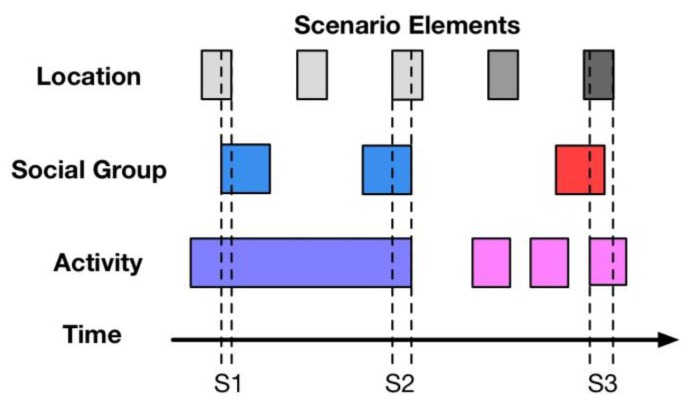
Description of all-elements-first algorithm.

The description of the All-Elements-First (AEF) Algorithm is shown in Figure 7.

As one scenario normalization method, the AEF algorithm ensures the correct rate of generated scenarios. That is to say, each scenario generated by the AEF algorithm is entirely different. The system can use this method securely without generating any repeated or incorrect scenarios. The process comprises three steps. In order to determine scenarios from a mass of scenario data accurately, the key point is to make sure of the start time and end time of each scenario. Hence, in the beginning of scenario normalization, the AEF algorithm would collect the start time and end time of each scenario element. Secondly, the time period which contains all the necessary scenario elements, i.e., the location, social group, and activity, is gathered successively. Finally, a new scenario is generated involving the detected scenario elements. For example, S1, S2 and S3, referring to scenario 1, scenario 2 and scenario 3, are detected according to the scenario elements, as shown in Figure 7.

#### 5.4.2. Elements Completion Algorithm

**Figure 8:**
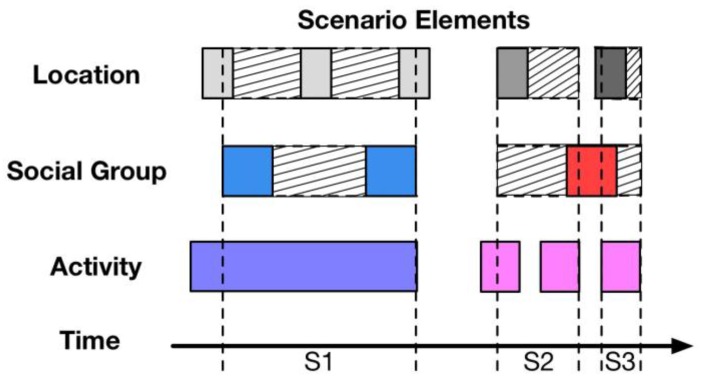
Description of elements completion algorithm.

The description of the Elements Completion (EC) algorithm is shown in Figure 8.

Although the AEF algorithm provides accurate scenarios, many scenarios that are not identified correctly, or scenarios have shortened time durations because of the inadequacy of scenario elements. Therefore, the EC algorithm is proposed correspondingly, to detect the potential scenarios as closely as possible according to the element completion. The core idea is to first use the EC algorithm to complete the missing elements, then use the AEF algorithm to detect scenarios. In comparison to the scenario generated by the AEF algorithm, the S1 illustrated in Figure 8 is much longer, since the location and social group is completed by the EC algorithm.

#### 5.4.3. Multiple Scenario Detection Algorithm

**Figure 9:**
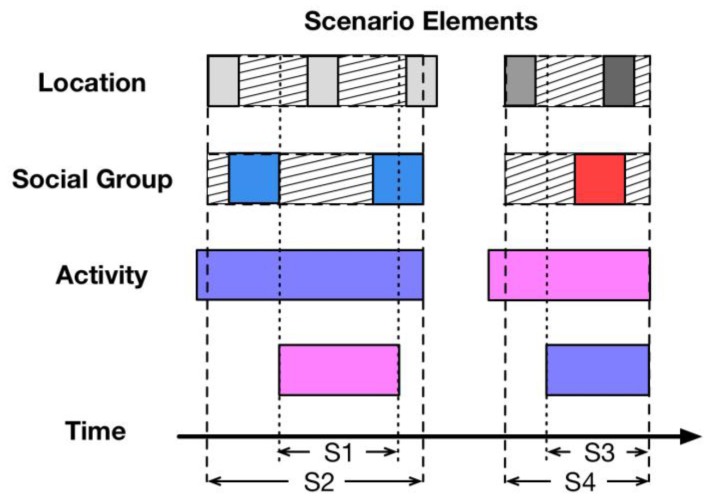
Description of multiple scenario detection algorithm.

The description of Multiple Scenario Detection (MSD) algorithm is shown in Figure 9.

The AEF algorithm and EC algorithm are prepared for single scenario detection. That means only a single scenario is detected during a period of time. However, sometimes a subject may do some activities simultaneously. For example, they may listen to music while working hard, or surf the Internet while chatting with a friend. Hence, the MSD algorithm is to detect multiple activities at the same time, and generate scenarios according to different activities in a period of time. After doing this, the MSD algorithm would use the EC algorithm for each activity respectively and generate into different scenarios. For example, the S1 and S2 or S3 and S4 are two pairs of scenarios within different time periods, as shown in Figure 9. The S1 and S2 are in the same time period, while the S3 and S4 are both within a different time period. Consequently, the EC algorithm can lengthen the detected scenarios easily.

## 6. Archetype Characterization

In this section, the description of psychological archetypes proposed in Psychology are given in sub-section one. However, such archetypes have no digital models for personality computing. To digitalize an archetype, the archetype classification is formulated the archetype elements. The final sub-section shows the process of archetype identification.

### 6.1. Archetype Description

As mentioned in Section 1, the term “archetype” refers to a human’s original pattern. According to the research of the psychologist, Carl Gustav Jung, the archetype is a universal and mythic character that exists within every human being. Concretely, Jung defined twelve primary types that symbolize basic human motivations. Each type has its own set of values, meanings and personality traits. The twelve archetypes are shown in Figure S1. As shown in Figure S1, the twelve archetypes are classified into four groups. For example, the archetype Lover belongs to the group “Connect to Others”. Meanwhile, each archetype has a word to indicate its feature. For example, the archetype Caregiver has the feature “Service”, while the archetype Hero has as its feature “Mastery”. According to Jung’s theory of personality, these twelve common archetypes are the basic components of a persona. As we mentioned, the persona is a person’s social mask. Therefore, a person in a certain situation would express one kind of persona, and such a persona would consist of one or several archetypes. For example, a person may show the archetype Hero and Caregiver at the same time when playing a card game because he wants to win but also wants to help his partner. Hence, the archetype seems to be the key to personality. Nevertheless, to the best of our knowledge, not much work has focused on the modeling of archetypes, and even less to personality computing according to archetypes. Therefore, it is critical for personality computing to digitalize the archetype.

The description of archetype Hero and Ruler are shown in Figures S2 and S3. Each archetype contains eight factors, namely a motto, core desire, goal, greatest fear, strategy, weakness, talent and the corresponding characters or roles. Some factors have a concrete description, such as the role of Hero, which includes the warrior, the soldier, the winner and the team player. However, some factors just have an abstract description, such as the motto of the Hero, which is “Where there’s a will, there’s a way”. For convenient archetype characterization, the role, goal, and strategy have been selected as the elements for archetype identification.

### 6.2. Archetype Measurement

This subsection presents the process of archetype measurement. Considering the obscureness of Jungian’s archetypes, the calculation of archetype simply based on the individual’s personal data seems to be opportunistic and feasible. Alternatively, the inquiry of archetypes directly on the individual’s part is more reliable and much easier than the former methods of archetype measurement. Faber and Mayer also select such method for the research of archetypes in media [36]. Hence, this research adopts the inquiry of archetypes based on the data of the user.

Prior to the measurement of archetypes, the tester should study and have a sound understanding of the definitions of archetypes, as showed in Table S2. When a scenario is normalized, the ABPC system would remind the tester to answer the question: “what kinds of archetypes are involved in your mind currently?”. The tester is required to reply to the system within 2 min. With this step finished, all of the archetypes with some correlation with the current scenario will be recorded.

In order to apply the archetype to the further modelling of persona, it is critical to formulate the archetype in advance. The archetype is classified as shown in Equation (6):

(6) $${\mathrm{Archetype}}_{{\mathrm{Scenario}}_{k}}=\left\{{\mathrm{ar}}_{1},{\mathrm{ar}}_{2},\ldots ,{\mathrm{ar}}_{n}\right\},$$

where each archetype ${\mathrm{ar}}_{k}$ belongs to the archetype set ${\mathrm{Archetype}}_{\mathrm{Scenario}}$ upon a certain Scenario, the ${\mathrm{Scenario}}_{k}$. The maximum number of n would not exceed 12, due to the sum of all archetypes are 12.

### 6.3. Archetype Classification

In this subsection, the archetype elements including role, goal and strategy are clarified separately with the following formulae, due to the description of each archetypes by Jung.

#### 6.3.1. Role

As one sign by which the archetype can be identified, role would be classified by analysing the social group in a scenario. The classification is shown in Equation (7):

(7) $$R=\left\{r_{1},r_{2},\ldots ,r_{n}\right\},$$

where each role element of $r_{k}$ belongs to the role set R, and $r_{k}$ refers to the relationship between a person and the k elements of his social group SG, that is $p_{k}$.

#### 6.3.2. Goal

The goal of each archetype can be inferred by the activity and emotion. For example, the goal of the archetype Innocent is to be happy, and if each time a person does the specific activity, i.e., watch a movie or listen to the music, he/she becomes happy, then he presents as the archetype Innocent. Generally, a person does some activities actively and his/her emotions change accordingly, which match a certain goal. The classification is shown in Equation (8):

(8) $$\mathrm{Goal}=\left\{g_{1},g_{2},\ldots ,g_{n}\right\},$$

where each goal $g_{k}$ belongs to the goal set Goal.

#### 6.3.3. Strategy

Similar to the goal being inferred by finding the pattern between activity and emotion, strategy is finding the features of behavior and goal. Concretely, strategy calculates the features of behavior and goal in terms of time cost, behavior incidence, etc. The classification is shown in Equation (9):

(9) $$\mathrm{STset}=\left\{{\mathrm{st}}_{1},{\mathrm{st}}_{2},\ldots ,{\mathrm{st}}_{n}\right\},$$

where each user’s strategy ${\mathrm{st}}_{k}$ is covered in the strategy set STset.

## 7. Persona Generation

As the social mask within the social interaction, the persona is the outermost layer of the personality structure. The correlation between the society and a person’s inner mind makes persona detection and generation possible. A description of persona is drawn in the next sub-section, and the persona construction method is illustrated in the last sub-section.

### 7.1. Persona Description

Our subject human being will show his/her persona during their interactions within society. For example, when surfing the Internet, watching movies, chatting with others, or even shopping in a shopping mall. Our state in terms of behavior, emotion, and physiology has different patterns when a person uses different personae. What’s more, different people display different personae to others even in an identical situation. For example, students will show totally different personae when they are listening to a lecture in the same classroom. Fortunately, the archetype theory proposed by Jung indicates a possible approach to variability of personae. The twelve typical archetypes exist universally in all human beings. In a certain situation, such as chatting with friends, people will show one or more archetypes during this situation. Hence, the persona in a certain situation could be regarded as the composition of one or more archetypes. Such archetypes are characterized in the last section, therefore in this section, the constituent elements of a persona should be clarified.

### 7.2. Persona Construction

According to the definition of persona by Jung, persona indicates “the relations between the individual consciousness and the society, fittingly enough a kind of mask, designed on one hand to make a definite impression upon others and on the other, to conceal the true nature of the individual.” Accordingly, such kind of a mask or image just shows a part of ourselves in a specific view or situation. Although the twelve universal archetypes are essential for one’s persona, each of which indicates a kind of model or a manner in dealing with the outer world, a certain persona may also display the individuals’ personality. Otherwise, people with the same archetype at the same situation would have the same persona. Apparently, since everyone has his/her unique image, or persona different from that of others, persona is diverse. Hence, persona consists of two essential parts, one is of which is the archetype and the other is the personality. According to Olsen’s construction of persona [37], persona should include information in the following categories:

- Persona’s Biographic Background
- Archetype
  ◆ Goal
  ◆ Role
  ◆ Strategy
- Personality Characteristics

The biographic background of persona contains the basic information that can’t be easily changed, such as the name and gender, the detailed description of which is shown in Table 1. The archetype is exactly the same as the formulation in Section 6. Notably, the personality characteristic refers to the detectable personality traits based on heterogeneous data sources, like the emotion stability, extraversion/ introversion. However, due to the diverse accessibility of personal data in different scenarios, the type and value of personality characteristics of different personae may vary.

## 8. Personality Modeling

As mentioned in Section 3, this section carries out a comprehensive personality analysis. The process of facet personae modeling and the personality computing based on such facet personae will be separately illustrated in the following two sub-sections.

### 8.1. Facet Persona Generation

As discussed in the last section, persona generated from a certain scenario shows a person’s characteristics during the social interaction, including the attitude, behavior pattern, goal, etc. Although there is a relative stability of a persona within a certain scenario, the shortage that such persona varies with its scenario has limited its usability for personality computing. Accordingly, it is necessary to find some of the general personae that cover multiple scenarios. For example, a person may hold a persona of “Orphan” and shows high tendency of introversion when he goes outside. Such general persona shared by numerous scenarios which happened outside are known as facet persona.

Sometimes, it’s simple to ascertain what kind of archetype a person presents according to his response. No problem occurs if a person just shows one archetype in a certain scenario either. The persona as a whole consists totally of such archetype. However, if a person replying with two or more archetypes simultaneously in a period of time, the appropriate way to measure a persona is to ascertain the proportion of each archetype during a certain scenario. The next sub-section details the algorithm to measure the proportion of each archetype when multiple archetypes exist in a certain scenario.

**Figure 10:**
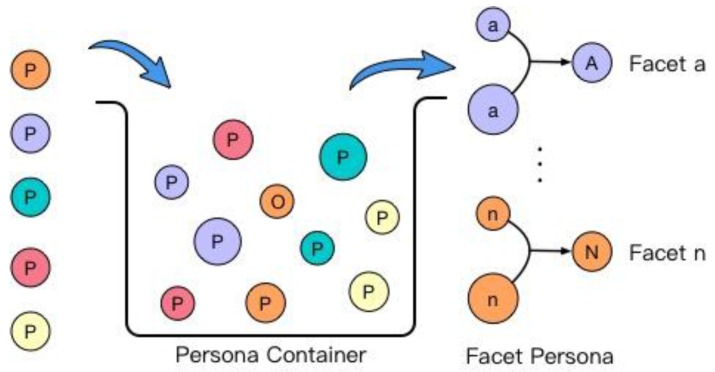
Process of facet personae generation.

A set of facet personae are generated entirely according to the personae from the persona container, as shown in Figure 10.

To achieve a comprehensive facet persona generation, every type of persona is put into a persona container in advance. As shown in Figure 10, the personae that satisfy the facet requirements are gathered together, and generate each kind of facet persona successively. Some examples of facet persona are shown in Table 2.

To calculate the proportion of each archetype, the formulation of the persona is necessary. Hence, the persona is classified as shown in Equation (10):

(10) $${\mathrm{Persona}}^{S}=\left\{{\mathrm{ar}}_{1},{\mathrm{ar}}_{2},\ldots ,{\mathrm{ar}}_{n}\right\},$$

where each archetype ${\mathrm{ar}}_{k}$ belongs to the persona ${\mathrm{Persona}}^{S}$. S refers to a certain scenario.

The fundamental idea is to calculate the identification incidence of each archetype, and then calculate relative proportions according to the incidence of each archetype. For example, the archetype Hero is identified three times from his/her personal data in a certain scenario, while the archetype Lover is identified twice. Therefore, the incidence of archetype Hero is 3, and the incidence of archetype Lover is 2. The expression for counting a concrete archetype is classified as shown in Equation (11):

(11) $${\mathrm{ar}}_{i}^{c}={\mathrm{Count}}_{i},$$

where the archetype ${\mathrm{ar}}_{i}^{c}$ upon a certain scenario c equals the ${\mathrm{Count}}_{i}$.

Subsequently, the proportion of a certain archetype refers to the proportion of its counts within the total counts of all archetypes. Hence, the proportion calculation is shown in Equation (12):

(12) $$P_{{\mathrm{ar}}_{i}}=\frac{{\mathrm{ar}}_{i}^{c}}{\sum_{k=1}^{n}{\mathrm{ar}}_{k}^{c}}$$

where the proportion of archetype ${\mathrm{ar}}_{i}$ is $P_{{\mathrm{ar}}_{i}}$ and is calculated by dividing the counts of archetype ${\mathrm{ar}}_{i}^{c}$ and the sum of archetype ${\mathrm{ar}}_{k}^{c}$.

### 8.2. Personality Computation

As mentioned in Section 3, the personality computation in this research is to summarize the character of the person, based on his facet personae modeled above. Therefore, the primary task of personality computation is to judge or evaluate the person, in accordance with the detectable personality trait from each facet persona. Facet persona merely contains a part of personality traits, as shown in the statistics pertaining to the frequency of each personality traits adopted in personality computing. Due to the continuous amendments of facet personae from the detected persona under different scenario, the approach to assess personality from facet personae can guarantee the accuracy of personality, compared with the result collected from questionnaire of personality. Considering the feasibility of this research, the personality computation would be simply carried out from the mean value of personality traits in each dimensions.

## 9. Experiments and Case Studies

In this beginning of this section, two fundamental issues are discussed with according experiments. They are (1) is Jung’s archetypes are universal and stable existence among different people, and (2) is the archetypes is varied with scenario. For the research in this paper on personality computing is founded on the Jung’s archetypes. Hence, the experiment of archetype classification based on the nomination of participants in different scenarios is performed firstly. After that, the features of the participants under different personae, including heartbeat, emotional stability and behavior, are compared with each other, so as to determine the availability and usability of the persona. Subsequently, case studies, such as scenario generation, facet personae modeling and personality, are presented respectively. In the end, the evaluation of personality trait is displayed.

### 9.1. Experiments

#### 9.1.1. Nomination of Archetypes

According to Jung’s definition, unconscious collectives contains twelve typical universal archetypes. That is to say, human beings would present more than one archetype in a certain scenario. The fundamental question is that whether the archetype in each scenario is stable and suitable. If only the archetype shows a sufficient level of stability in the certain scenario, it is possible for the system to measure the corresponding persona using digital means. Therefore, in this research, the main target is to show the relatively stable existence of archetypes in the certain scenario among different people using anonymous nomination.

In this sub-section, the archetypes of 60 participants from several scenarios are collected, as shown in Table 3. These participants are required to familiarize themselves with the definition of each archetypes before the test. Afterwards, each participant is required to vote to the most suitable archetypes in such scenario, based on his or her own opinion. Specifically, five normal scenarios are selected, which are: 1. make a presentation; 2. chat with friend; 3. work alone at home; 4. have a seminar with a teacher or a supervisor; and 5. shopping at the supermarket. The number of each column represents the number of votes for each archetype. What is worth noting is that the maximum number of archetype for one scenario cannot exceed 3. The numbers marked in red in Table 3 show the most common archetypes shared by numerous individuals. Most people would show the Orphan IN the presentation. Multiple archetypes are displayed when they are chatting with friends. Moreover, the archetype Jester would make appearance more frequently when a person goes shopping at a supermarket. These results demonstrated the existence of the most common archetype in some regular scenarios.

#### 9.1.2. Visualization of Characteristic

**Figure 11:**
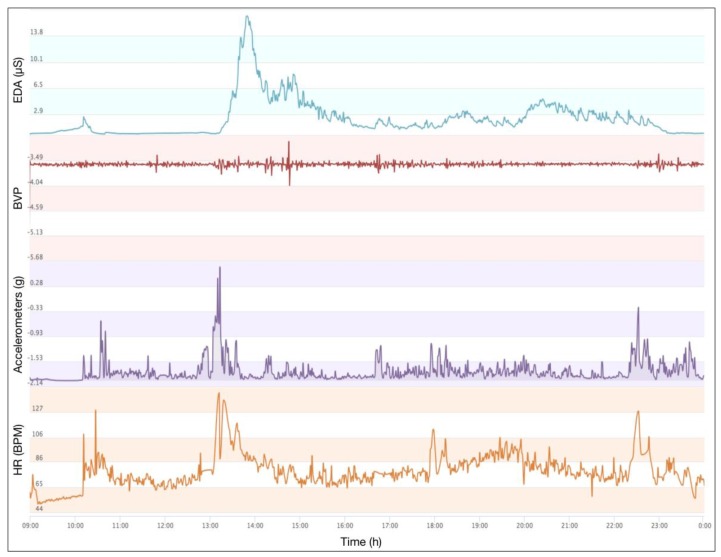
An example of wearable sensors’ data.

In this sub-section, the visualization of a person’s characteristic is shown, based on/without his or her persona. Three features are selected from the experiment, based on the long-term personal data of the participants. The aim of this experiment is to show the stability of a certain characteristic in a specific persona, and to demonstrate the disorder of the same characteristic ignored in the persona. The participants need to wear the Empatica E4 wristband for 10 days. An example of the sensors’ data from such wristband are shown in Figure 11. The electrodermal activity (EDA) is selected to measure the autonomic nervous system activity from the continuous variation in the electrical characteristics of the skin. EDA is highly responsive to emotions. Fear, anger, startled response, orienting response, etc., are among the reactions that may be reflected in EDA. Hence, in this research, the emotional variation of the participant can be sensed and recorded in the EDA data. Meanwhile, the delta acceleration from accelerometers is selected to indicate the behavior variation of the participants. Heart Rate data is also selected to reveal the physiological features in the course of different persona.

**Figure 12:**
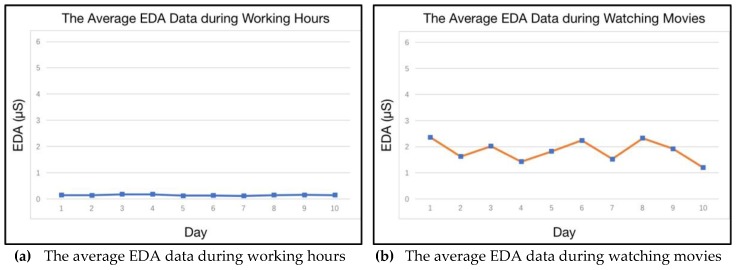
The daily average emotional response during different scenarios.

**Figure 13:**
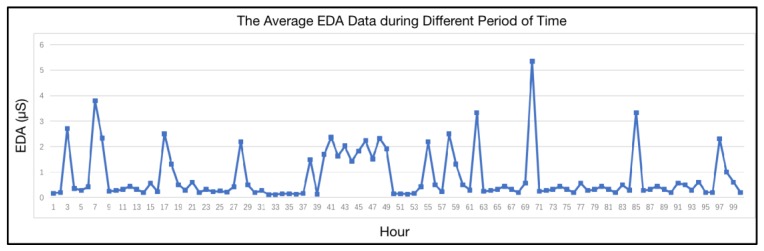
The emotional response without scenario classification.

The experiment in this sub-section demands that the participants should join in some particular scenarios with the same activities every day. In this experiment, two daily scenarios are selected accordingly. The participants work alone and they watch movie. The daily average emotional response of two scenarios are drawn in Figure 12. Apparently, the emotional response shows a higher level of stability during working hours. As shown in the curve in the right side of Figure 12, emotion shows regular variations when the participants are watching movie. As one of personality traits, the stable existence of the two kinds of emotion can be found in different scenarios. This result indicates that there are two different personae with the participants when they are working and when they are watching movies. Additionally, a whole day’s emotional response is shown in Figure 13. The curve suggests a low level of emotional stability, regardless of the classification of scenarios.

**Figure 14:**
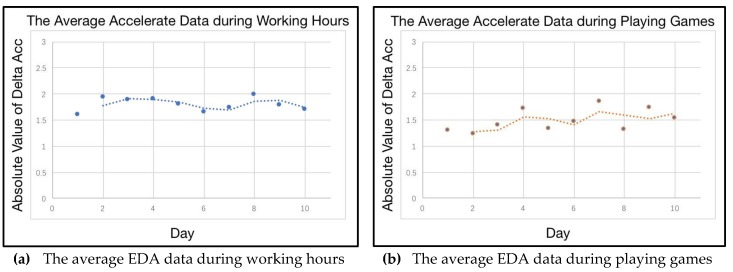
The average degree of activity during different scenarios.

In addition to the visualization of emotional response, Figure 14 displays the degree of activity in different scenarios as well. Personae, no matter of working and playing games, show a high level of consistency when the participants join in this activity. This pattern is revealed in the two fitted curves in Figure 14.

### 9.2. Case Studies

#### 9.2.1. Case Study on Scenario Generation

**Figure 15:**
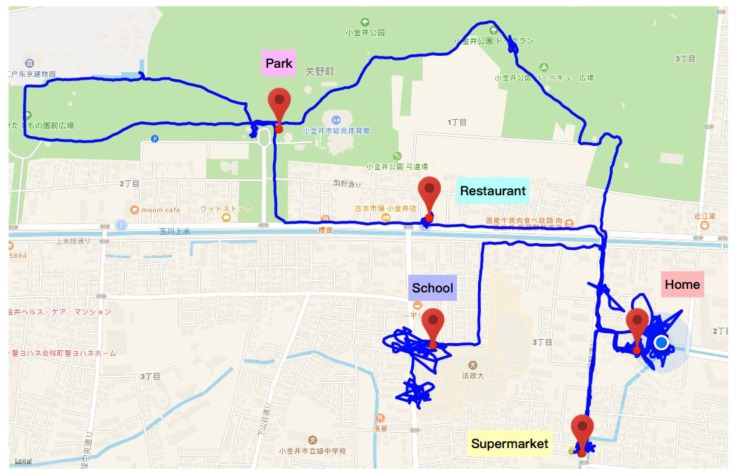
GPS data from smartphone.

**Figure 16:**
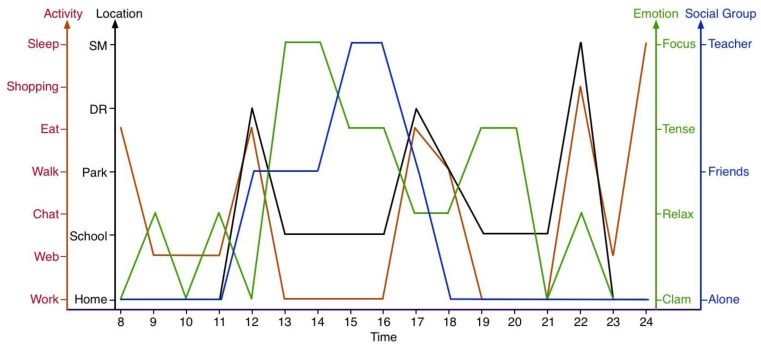
Scenario data in a whole day’s Life.

The scenario elements were collected separately according to a tester’s smartphone data, wearables data and Internet data. Specifically, the location was detected by GPS data from smartphone, as shown in Figure 15. Five regular places are home, school, a restaurant, a park and a supermarket. And the scenario data of a whole day of life are displayed in Figure 16.

#### 9.2.2. Case Study on Facet Personae Construction

**Figure 17:**
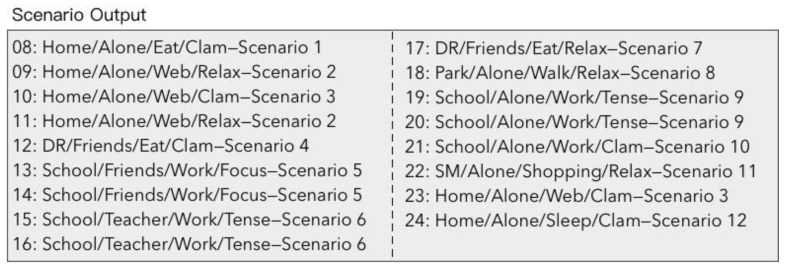
Scenario output.

**Figure 18:**
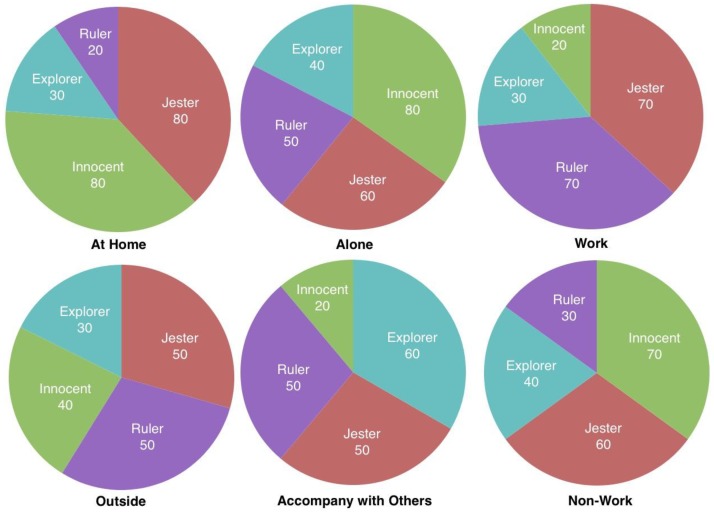
Results of facet personae.

As the fundamental part for personality computing, the facet persona shows the different composites of archetypes in different situation. Due to the measurement of persona through various personal data, the facet persona in terms of three different facets are classified accordingly, and the results shown in Figure 17. As components of persona, four archetypes were characterized, namely, Jester, Innocent, Explorer, and Ruler. The four archetypes were generated in different proportion into each kind of persona in the twelve scenarios above. Three different facets were selected, referring to a tester being home or not, alone or not, or working or not. A group of facet personae are shown in Figure 18.

### 9.3. Comparison between Personality Computing and Personality Questionnaire

As a comparison, experiments to verify the stability of personality traits were also performed, as shown in Table 4. Four testers did the BFI-10 (Big Five Inventory-10) questionnaire twice on different days. Op, Co, Ex, Ag and Ne show the five traits of Big Five personality. While AE refers to the absolute error of its corresponding element. For example, the highest absolute error was 14%, indicating that Neuroticism, as one of Tester Ds’ personality traits, was unstable. In comparison, the result of persona showed several stable compositions of human archetypes in different facets. Lifestyle in different facets is presented clearly in this research.

## 1.0. Conclusions and Future Work

### 10.1. Conclusions

In this paper, we have proposed an archetype-based modeling of persona to achieve three main objectives. The first objective was to detect scenarios according to user behavior effectively and swiftly. To that end, we implemented a specific scenario model with a classification algorithm for each corresponding scenario element. Each of the three fundamental elements, comprising location, social group and user activity, are described in the situation along with its essential information during an event. Therefore, this essential information is properly prepared for analysis of a user’s mental state. However, due to the difficulty of modeling in the analysis of the user’s mental state, the second objective was to achieve a model with a comprehensive individual description, based on a scenario generated previously. To reach a more detailed level of description, a conceptual model called persona was applied in this research accordingly, taking into account the fact that the persona model merely depicts the user model from one certain aspect, for example, the behavior trait used in Facebook. Hence, the second objective was to rebuild the persona based on psychological theory, i.e., Jung’s theory of personality. According to this theory, twelve archetypes common to all human beings are selected for the construction of a persona. Meanwhile, taking the personal physiological state, emotion state and behavior state into consideration, the presented archetypes of a person involved in a certain scenario were detected. The persona modeling mainly focused on determining the proportion each archetype presented in a given scenario.

The third objective was to create a set of personae called facet personae to generate a complete individual description under different circumstances. The facet personae are a set of personae indicating the user’s states in different facets, i.e., alone or accompanied by others. In addition to this conceptual model of persona, a modeling mechanism was illustrated to depict the appropriate process generating personality from those facet personae.

### 10.2. Future Work

However, there are three kinds of work that remain to be done in future studies. Firstly, more elements in scenario detection need to be taken into consideration for the construction of facet personae modeling. Secondly, the facet personae modeling function needs to be further improved to serve the development of mature modeling mechanisms. Third, a wide range of experiments, especially the normalization of several common scenarios applicable to a variety of people and the facet personae modeling based on such scenarios, need to be carried out for further analysis and evaluation.

## Figures and Tables

**Table 1:** Persona’s biographic background.

Elements 1	Description
Name	A name is a term used for identification
Gender	The distinction between male and female
Nationality	A legal relationship between a person and a state
Occupation	A person’s role in society

**Table 2:** Examples of facet personae.

Facet List	Description	Elements
Study	Classified the facet according to the activity type	● Archetypes ● Proportion of Archetypes ● Goal ● Role ● Personality Trait
Work		
Entertainment		
Face the Friend	Classified the facet due to the person accompany with	
Face the Family		
Face the Boss		
Within Internet	Classified the facet due to with or without the Internet	
Without Internet		
At Home	Classified the facet due to location data	
At Public Occasions		
At Work Place		
Use Smartphone	Classified the facet due to the used device	
Use Computer		
In the Morning	Classified the facet due to time period of a day	
In the Afternoon		
At Night		

**Table 3:** Archetype nomination frequencies.

Scenario/Archetype	Number of Nominations												N
	IN	OR	HE	CA	EX	RE	LO	CR	JE	SA	MA	RU	
**Presentation**	10	6	38	2	14	6	0	14	2	15	2	9	60
**Chat with Friend**	31	32	4	36	4	3	8	0	14	4	1	1	60
**Work Alone at Home**	10	10	10	2	16	13	3	15	11	14	2	1	60
**Seminar with Boss**	12	12	15	7	14	14	4	14	0	16	13	0	60
**Shop at Supermarket**	16	0	0	8	16	2	5	5	33	2	3	6	60
**Average**	15.8	12	13.4	11	12.8	7.6	4	9.6	12	10.2	4.2	3.4	60

IN, Innocent; OR, Orphan; HE, Hero; CA, Caregiver; EX, Explorer; RE, Rebel; LO, Lover; CR, Creator; JE, Jester; SA, Sage; MA, Magician; RU, Ruler.

**Table 4:** Four testers’ result of BFI-10 questionnaire.

	OP	Co	Ex	Ag	Ne
**Tester A**	68%	48%	58%	73%	35%
	63%	68%	60%	78%	45%
**AE**	2.5%	**10.0%**	**1.0%**	2.5%	6.5%
**Tester B**	58%	55%	33%	95%	35%
	58%	40%	40%	90%	30%
**AE**	**0.0%**	**7.5%**	3.5%	2.5%	2.5%
**Tester C**	73%	63%	40%	70%	38%
	53%	53%	50%	65%	53%
**AE**	**10.0%**	5.0%	5.0%	**2.5%**	**2.5%**
**Tester D**	70%	80%	78%	75%	48%
	70%	80%	65%	80%	20%
**AE**	**0.0%**	**0.0%**	6.5%	2.5%	**14.0%**
**MAE**	3.125%	5.625%	4.0%	2.5%	6.375%
Note: The bold items were the maximum or minimum absolute error (AE). Legend: Op—Openness; Co—Conscientiousness; Ex—Extraversion; Ag—Agreeableness; Ne—Neuroticism.					

## Supplementary Materials

The following are available online at http://www.mdpi.com/1424-8220/18/3/684/s1. Figure S1: Jung’s 12 Archetypes, Figure S2: Description of the Hero Archetype, Figure S3: Description of the Ruler Archetype, Table S1: Heterogeneous Data Sources, Table S2: Archetype Definitions.
