# Supplementary material for: Archetype-Based Modeling of Persona for Comprehensive Personality Computing from Personal Big Data
Source: Sensors (Basel). 2018 Feb 25;18(3):684. doi: 10.3390/s18030684 (PMC5876706; doi:10.3390/s18030684)
Supplement: Supplementary file 1 [file sensors-18-00684-s001.pdf]

sensors

Article

Archetype-Based Modeling of Persona for Comprehensive Personality Computing from Personal Big Data

Ao Guo [1,*] **and Jianhua Ma** [2]

[1] Graduate School of Computer and Information Sciences, Hosei University, Tokyo 184-8584, Japan;
[2] Faculty of Computer and Information Sciences, Hosei University, Tokyo 184-8584, Japan; jianhua@hosei.ac.jp
* Correspondence: guo.ao.33@stu.hosei.ac.jp or guoaokiral@gmail.com; Tel.: +81-70-1395-0028

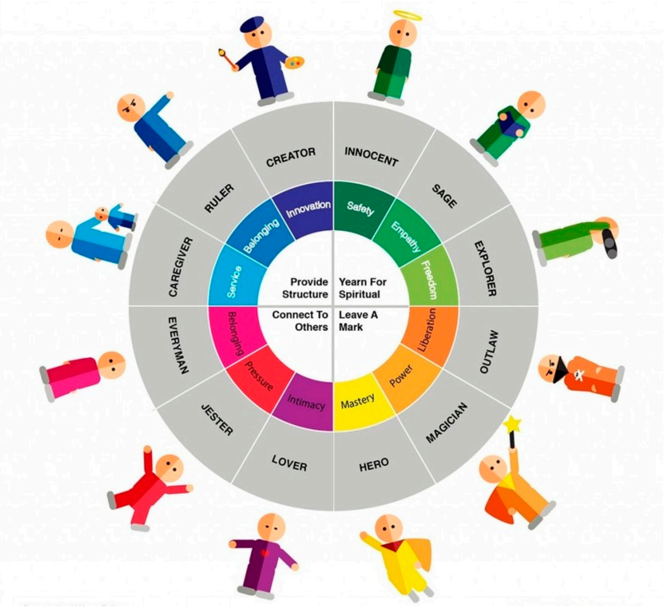

Figure S1: Jung's 12 Archetypes.

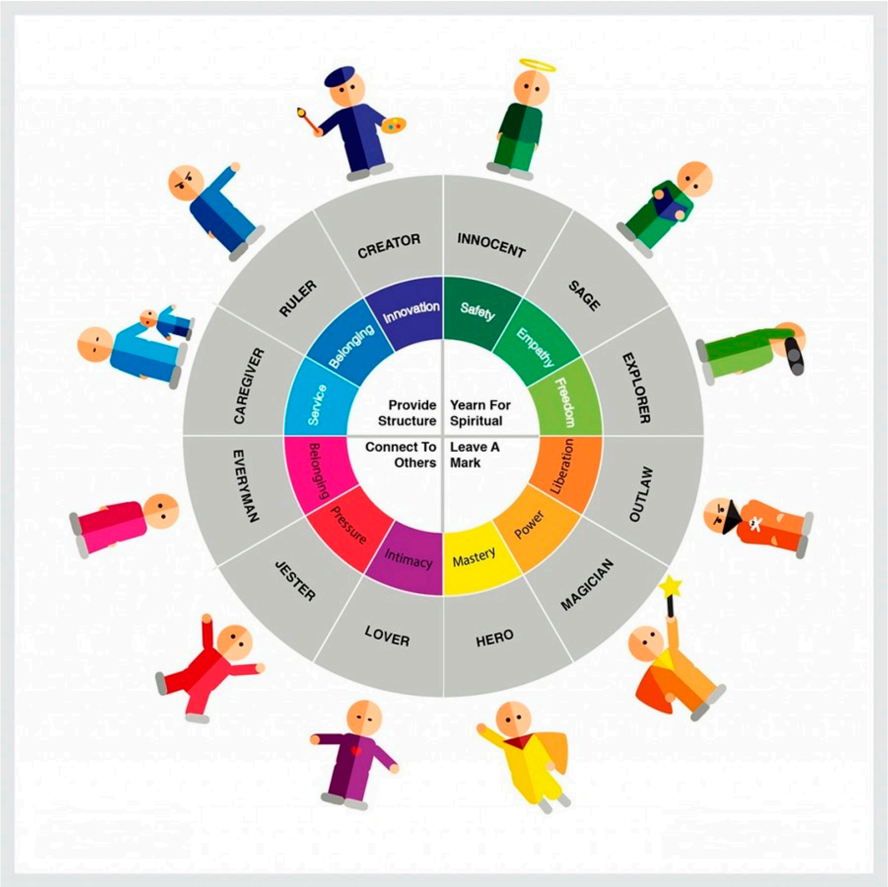

Figure S2: Description of the Hero Archetype.

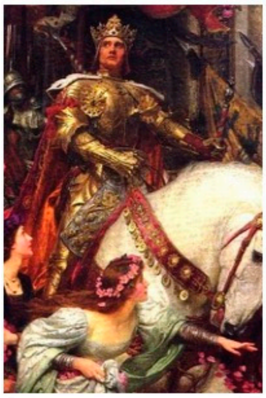

12. **The Ruler**
Motto: Power isn't everything, it's the only thing.
Core desire: control
Goal: create a prosperous, successful family or community
Strategy: exercise power
Greatest fear: chaos, being overthrown
Weakness: being authoritarian, unable to delegate
Talent: responsibility, leadership
The Ruler is also known as: The boss, leader, aristocrat, king, queen, politician, role model, manager or administrator.

Figure S3: Description of the Ruler Archetype.

Table S1: Heterogeneous Data Sources.

Data Source	Name	Unit	Time Unit
Smartphone Sensor	GPS Data	(Lng, Lat)	s
Smartphone Sensor	Angular Acceleration	(Ax, Ay, Az)	s
Smartphone APP	Step Counter	Step	m
Smartphone Sensor	Barometer	S	s
Smartphone APP	Energy Burn	Calorie	m
Smartphone APP	Step Distance	Meter	m
Smartphone Sensor	Voice Record	C	ms
Smartphone Sensor	Image Record	C	ms
Smartphone Sensor	Acceleration	(Gx, Gy, Gz)	ms
Smartphone Sensor	Temperature	℃	s
Smartphone Sensor	Light	Lux	ms
Smartphone Sensor	Humidity	%	s
Smartphone Sensor	Proximity	cm	s
GPS Tracker	GPS Data	(Lng, Lat)	ms
iWatch	GPS Data	(Lng, Lat)	s
Mindwave	Mind Wave	(Alpha, Beta, Gamma)	ms
iWatch	Heart Rate	Count	m
Smart Shoes	Step Counter	Count	ms
Hitoe C3Fit	Heart beat	Rate	ms
Spire	Breathing Rate	Count	m
Spire	Emotion	Rate	m
Smart Ring	Acceleration	(Gx, Gy, Gz)	ms
Fitbit Charge 2	Sleep Data	Sleep Hour	h
Empatica E4	EDA Data	Emotion Response	μs
Empatica E4	BVP Data	Blood Volume Pulse	ms
Myo	Gesture	(Gx, Gy, Gz)	s
Myo	Acceleration	(Gx, Gy, Gz)	ms
Jins Meme	Eye Focus	(Gx, Gy, Gz)	s
Jins Meme	Eye Tracker	(X, Y)	s
Qardio Arm	Blood Pressure	(H, L)	s
Internet	Web History	Site	s
Internet	Facebook State	Post	s
Internet	Wechat Message	Message	s
Internet	Wechat Moment	Post	s
Internet	Shopping data	Records	s
Internet	Twitter State	Post	s

Table S2: Archetype Definitions.

Archetype	Definition
Caregiver	Represented by caring, compassion, and generosity. Commonly protective, devoted, sacrificing, nurturing, and often parental. Usually very benevolent, friendly, helping, and trusting
Creator	Represented by the innovative, the artistic, and the inventive. Often non-social; perhaps a dreamer; looking for novelty and beauty and an aesthetic standard. Will emphasize quality (over quantity), being highly internally driven
Everyman/ Everywoman	Represented by the working-class common person; the underdog; the neighbor. Persevering, ordered, wholesome; usually candid and sometimes fatalistic. Often self-deprecating; perhaps cynical, careful, a realistic and often disappointed humanist
Explorer	Represented by an independent, free-willed adventurer. Seeks discovery and fulfillment. Often solitary; spirited and indomitable; observer of the self and environment. Constantly moving; a wanderer
Hero	Represented frequently by the courageous, impetuous warrior. Noble rescuer and crusader; must often undertake an arduous task to "prove their worth" and later become an inspiration. Symbolically the "dragonslayer"—the redeemer of human strength
Innocent	Represented by the pure, faithful, naive, childlike character. Humble and tranquil; longing for happiness and simplicity—a paradise. Often a traditionalist; saintly; symbolizing renewal
Jester	Represented by living for fun and amusement; a playful and mischievous comedian. Usually ironic and mirthful, sometimes irresponsible; a prankster. Enjoys most a good time and diversion from care
Lover	Represented by the intimate, romantic, sensual, and especially passionate. Seeking mainly to find and give love and pleasure. Seductive and delightful, but perilous—often tempestuous and capricious. Often a warm, playful, erotic, and enthusiastic partner
Magician	Represented by the physicist; the visionary; the alchemist. Seeking the principles of development and how things work; a teacher, a performer or a scientist. Fundamentalist interested in natural forces, transformations, and metamorphoses
Outlaw	Represented in the rebellious iconoclast; the survivor and the misfit. Often vengeful, a disruptive rule-breaker, possibly stemming from hidden anger. Can be wild, destructive and provoking from a long time spent struggling or injured
Ruler	Represented by a strong sense of power and control: the leader; the boss; the judge. Highly influential, stubborn, even tyrannical. Maintains a high level of dominance; can apply to an administrator, arbiter, or a manager of others
Sage	Represented by a valuing of enlightenment and knowledge; truth and understanding. This is the expert and the counselor, possessing wisdom and acumen, perhaps a bit pretentious. Scholarly, philosophical, intelligent; a mystical and prestigious guide in the world
Shadow	Represented by the violent, haunted, and the primitive; the darker aspects of humanity. Often seen in a tragic figure, rejected; awkward, desperately emotional. Can be seen to lack morality; a savage nemesis
